# New superconductor Li_*x*_Fe_1+δ_Se (*x* ≤ 0.07, *T*_c_ up to 44 K) by an electrochemical route

**DOI:** 10.1038/srep25624

**Published:** 2016-05-11

**Authors:** Anastasia M. Alekseeva, Oleg A. Drozhzhin, Kirill A. Dosaev, Evgeny V. Antipov, Konstantin V. Zakharov, Olga S. Volkova, Dmitriy A. Chareev, Alexander N. Vasiliev, Cevriye Koz, Ulrich Schwarz, Helge Rosner, Yuri Grin

**Affiliations:** 1 Department of Chemistry, Lomonosov Moscow State University, 119991 Moscow, Russia; 2 MPG-MSU Partner Group, Department of Chemistry, Lomonosov Moscow State University, 119991 Moscow, Russia; 3 Department of Physics, Lomonosov Moscow State University, 119991 Moscow, Russia; 4 Institute of Experimental Mineralogy, Russian Academy of Sciences, 142432 Chernogolovka, Russia; 5 Max-Planck-Institut für Chemische Physik fester Stoffe, 01187 Dresden, Germany

## Abstract

The superconducting transition temperature (*T*_c_) of tetragonal Fe_1+δ_Se was enhanced from 8.5 K to 44 K by chemical structure modification. While insertion of large alkaline cations like K or solvated lithium and iron cations in the interlayer space, the [Fe_2_Se_2_] interlayer separation increases significantly from 5.5 Å in native Fe_1+δ_Se to >7 Å in K_*x*_Fe_1−*y*_Se and to >9 Å in Li_1−*x*_Fe_*x*_(OH)Fe_1−*y*_Se, we report on an electrochemical route to modify the superconducting properties of Fe_1+δ_Se. In contrast to conventional chemical (solution) techniques, the electrochemical approach allows to insert non-solvated Li^+^ into the Fe_1+δ_Se structure which preserves the native arrangement of [Fe_2_Se_2_] layers and their small separation. The amount of intercalated lithium is extremely small (about 0.07 Li^+^ per f.u.), however, its incorporation results in the enhancement of *T*_c_ up to ∼44 K. The quantum-mechanical calculations show that Li occupies the octahedrally coordinated position, while the [Fe_2_Se_2_] layers remain basically unmodified. The obtained enhancement of the electronic density of states at the Fermi level clearly exceeds the effect expected on basis of rigid band behavior.

Tetragonal iron selenide, Fe_1+δ_Se (0.01 ≤ δ ≤ 0.04), is one of the most interesting representatives of iron-based superconductors discovered a few years ago^1^,^2^,^3^. Although the superconducting transition temperature is rather low (*T*_c_ = 8.5 K for δ = 0.01), Fe_1+δ_Se offers unique opportunities for structure modification which significantly enhances *T*_c_ up to 40 K^4^,^5^,^6^,^7^,^8^,^9^. The increase of *T*_c_ is accompanied by changes of distances and interactions between the anti-fluorite layers [Fe_2_Se_2_]: adjacent [Fe_2_Se_2_] layers shift by (½, ½, 0) and the interlayer space is filled by heavy alkali metals^4^,^5^ or solvated metal cations (chemical intercalation using anoxic polar solvent^6^,^7^). Very interesting results were obtained in hydrothermal synthesis experiments which yielded the formation of Li_1−*x*_Fe_*x*_(OH)Fe_1−*y*_Se with *T*_c_ ≈ 40 K (refs 8,9). The Li_1−*x*_Fe_*x*_(OH)Fe_1−*y*_Se structure is a stacking arrangement of alternating [Fe_2_Se_2_] and [(Li,Fe)OH] layers, the latter acting as charge reservoirs^9^.

The literature information on electrochemical studies of iron chalcogenides is limited to just a few papers. So, the electrochemical Li-ion intercalation into FeSe_1−*x*_Te_*x*_ results in the formation of Li_*y*_FeSe_1−*x*_Te_*x*_ for 0.25 ≤ *x* ≤ 1 (ref. 10). However, the observed preservation of lattice parameters and *T*_c_ (even at high *y* values) are not consistent with the claimed formation of the Li-intercalated phases. Moreover, the structural characterization is insufficient. Carbon-coated FeSe-nanoparticles were investigated as an anode material for lithium-ion batteries with a sustainable reversible capacity of 340 mAh·g^−1^ (ref. 11). The reversible charge-discharge cycling implicates the amorphization of the initial iron selenide and probably involves the formation of metallic Fe-nanoparticles and Li_2_Se. Successful electrochemical intercalation of potassium and sodium into FeSe has been reported very recently^12^.

In this work we have successfully modified superconducting properties of Fe_1+δ_Se for the first time by electrochemical lithium intercalation. This approach allows the preservation of the native structure and the enhancement of *T*_c_ up to 44 K due to changes of the carrier concentration. The obtained results provide the opportunity to shed new light on the mechanism of superconductivity in Fe-based superconductors and opens new ways for enhancing *T*_*c*_. The detailed investigation of the electrochemical behavior of Fe_1+δ_Se upon lithium intercalation conditions clarifies the existing contradictions and demonstrates the potential of the electrochemical approach to modify crystal structure and properties of Fe_1+δ_Se.

## Results and Discussion

Electrochemical intercalation of Li-ions into tetragonal Fe_1+δ_Se was performed using a single-phase polycrystalline sample and large aggregates of single crystals. The polycrystalline sample consisted of agglomerates of particles with linear sizes between 1 and 10 μm (Fig. SI1). The lattice parameters *a* = 3.7720(2) Å and *c* = 5.5231(4) Å obtained from powder X-ray diffraction data (PXRD, Fig. SI2) are in good agreement with the literature^13^,^14^. According to the SEM study, crystal aggregate of tetragonal Fe_1+δ_Se with a linear size of approximately 1 × 2 mm contains oriented single-crystal blocks (Fig. SI3).

**Figure 1:**
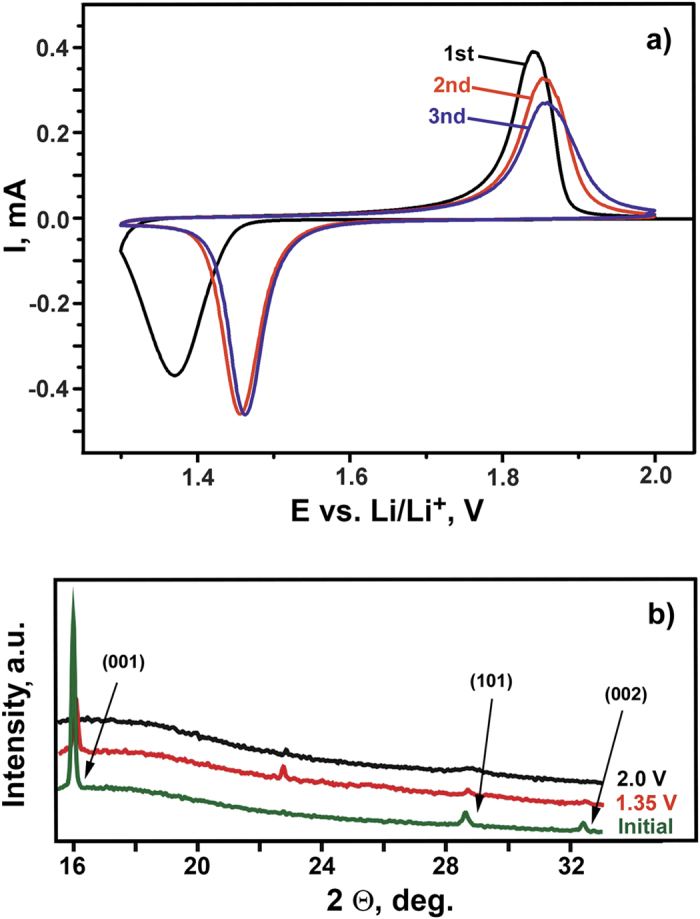
(**a**) CVAs curves (first three cycles) obtained for the polycrystalline Fe_1+δ_Se electrode in the potential range 1.3−2.0 V *vs*. Li/Li^+^ (scan rate of 0.05 mV·s^−1^); (**b)** a part of *ex-situ* PXRD patterns for polycrystalline Fe_1+δ_Se electrodes at different potentials during 1st cycle. Reflections of tetragonal Fe_1+δ_Se are indexed. The diffraction maximum at 2*θ* of approx. 23° is unidentified. Electrochemical investigations at the same conditions (potential region, scan rates) revealed the appearance of this maximum also in the patterns of electrodes based on other active materials and even of the ‘idle’ electrode. Thus, the maximum is assigned to products of the electrolyte reduction occurring at the effective electrode surface.

Cyclic voltammetry (CVA) studies of polycrystalline Fe_1+δ_Se electrodes between 1.3 V and 2 V *vs.* Li/Li^+^ (Fig. 1a) revealed the occurrence of a redox process with a significant reversible capacity (*ca*. 300 mAh·g^−1^). In the 1^st^ cycle the reduction peak is at *ca*. 1.3 V, the oxidation peak is at *ca*. 1.85 V. Upon cycling, the potential of the reduction peak shifts towards more positive potential values (1.4–1.5 V), while the position of the corresponding oxidation peak does not change considerably, although a significant broadening of the peak occurs. *Ex-situ* PXRD data for the electrode material charged or discharged at different potential values show a drastic decrease of the reflections intensities of Fe_1+δ_Se without noticeable lattice parameters variation. No evidence for new crystalline phases is observed (Fig. 1b). These findings are compatible with an irreversible phase transformation.

The observed capacity value suggests irreversible decomposition of iron selenide in the 1^st^ cycle according to reaction equation (1). This assignment is also in agreement with earlier conclusions^11^. We assume that this reaction results in the formation of iron and lithium selenide nanoparticles which could not be detected by our PXRD measurements.


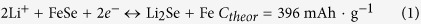


**Figure 2:**
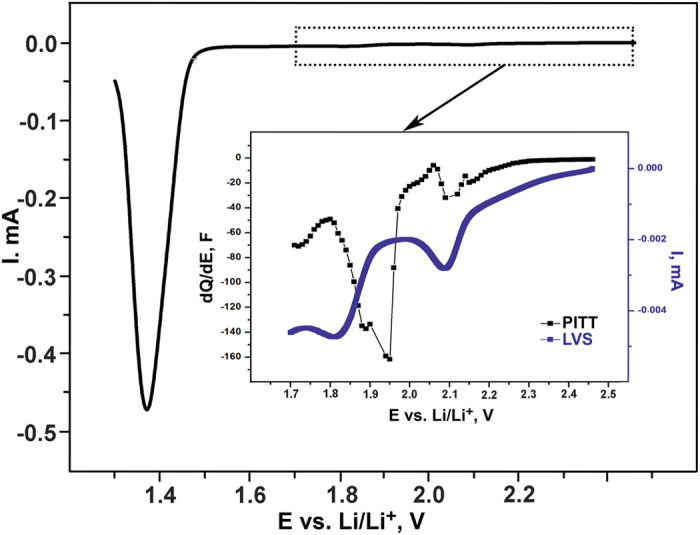
LVS curve obtained for the polycrystalline Fe_1+δ_Se electrode (scan rate 0.02 mV·s^−1^, 2.5–1.2 V *vs.* Li/Li^+^). The inset shows the LVS curve and the differential capacity obtained by PITT in the potential range 2.5–1.7 V *vs.* Li/Li^+^ in detail.

The reduction process was studied in more detail by means of slow scan-rate linear voltammetry (LVS) and potentiostatic intermittent-titration technique (PITT) combined with chronoamperometry (CA). This study reveals the complex character of the 1^st^ reduction curve including additional peaks at *ca*. 2.1, 1.8 and 1.6 V *vs* Li/Li^+^ (Fig. 2, Fig. SI4) preceding the main conversion peak (starting at *ca.* 1.5 V), similarly to previous observations^11^. The results of analogous investigation of carbon-free electrodes allowed attributing the rather intense peak at *ca*. 2.1 V to the electrolyte reduction at the carbon surface (Fig. SI4). The charge passed through the cell in the potential region of 3.0−1.55 V (before the major reduction peak) corresponds to *ca*. 11 mAh·g^−1^ (0.055 Li^+^ per f.u.). Further application of CA at 1.7 V for a few days did not result in significant growth of the current. This voltage is between the peaks of the assumed Li^+^ ion intercalation and the main conversion process in the differential capacity plot (Fig. 2). We suppose that the amount of the intercalated Li in Li_*x*_Fe_1+δ_Se at this voltage corresponds to *x* ≈ 0.06. According to PXRD, the Fe_1+δ_Se phase is preserved in the PITT procedure. Even after long CA treatment at 1.7 V (several days up to a week) all samples exhibit the same behavior: after turning-off the electrochemical cell, the potential increases rather rapidly from 1.7 V to approx. 2−2.5 V *vs.* Li/Li^+^ as in case of initial Fe_1+δ_Se. We regard this effect as the result of the spontaneous decomposition of the intercalated phase Li_*x*_Fe_1+δ_Se. Indeed, magnetic measurements of the electrode treated at 1.7 V vs. Li/Li^+^ and kept inside the glovebox at room temperature for several days showed the presence of the initial Fe_1+δ_Se phase only.

**Figure 3:**
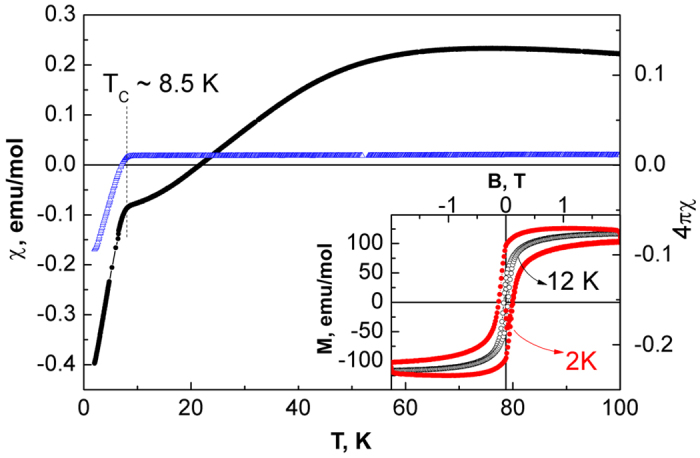
Temperature dependence of the magnetic susceptibility of the initial polycrystalline Fe_1+δ_Se electrode (blue open curve) and of the polycrystalline Fe_1+δ_Se electrode treated at 1.7 V *vs* Li/Li^+^ (black solid spheres) measured in the ZFC regime at 0.01 T. The inset represents magnetization curves measured at 2 K and 12 K.

Magnetic measurements of the polycrystalline Fe_1+δ_Se electrode treated with LVS and CA at 1.7 V *vs*. Li/Li^+^ unveiled drastic changes in comparison with native Fe_1+δ_Se (Fig. 3). The temperature dependence of the magnetic susceptibility χ(T) shows a strong diamagnetic signal at low temperatures with a kink at about 8 K and a broad maximum at about 70 K. The pronounced, negative slope of the magnetic susceptibility at low temperature indicates the presence of initial superconducting tetragonal Fe_1+δ_Se^3^. The broad maximum at high temperatures signals the presence of several percent of elemental iron in the system, presumably in nano-structured form^15^. This attribution is strongly supported by magnetization curves measured at 2 and 12 K, see inset of Fig. 3. Both curves evidence hysteresis loops which are typical for iron nanoparticles below the blocking temperature^15^,^16^. Besides, the *M*(*H*) dependence measured at 2 K is decorated with a weak diamagnetic response seen in low fields due to the presence of the superconducting phase. Thus, the redox process between 1.8 V and 1.6 V *vs* Li/Li^+^ is characterized by a very small capacity value and accompanied by the conservation of the Fe_1+δ_Se structure. The observed capacity value corresponds to a small amount of Li reacted with Fe_1+δ_Se and being intercalated as Li^+^. The spontaneous potential increase after cell turning-off together with the magnetic measurements data can be understood taking into account the instability of the product of the electrochemical redox process if the potential is not applied.

**Figure 4:**
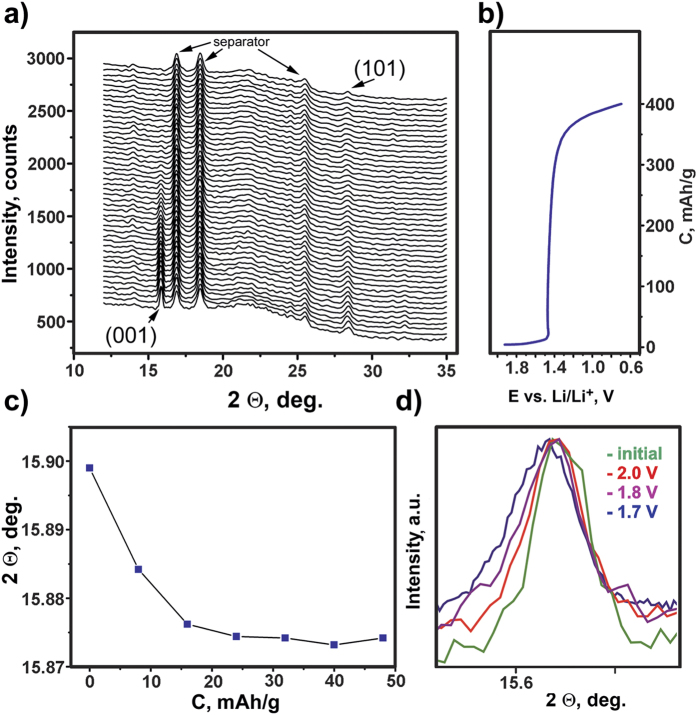
*In-situ* X-ray powder diffraction experiment performed for the polycrystalline Fe_1+δ_Se electrode during galvanostatic charge with potential limitation (approx. C/20 rate, 2.0−0.7 V *vs.* Li/Li^+^). (**a)** obtained PXRD patterns (reflections of the tetragonal Fe_1+δ_Se are indexed, additional observed maxima correspond to the components of the *in-situ* electrochemical cell); (**b)** *E*−*C* curve corresponding to the PXRD patterns; (**c)** dependence of *001* reflection position on the passed charge; (**d)** transformation of the *001* reflection at different potentials under potentiostatic conditions.

To clarify the behavior of Fe_1+δ_Se in the applied electrochemical potentials, *in-situ* PXRD studies in galvanostatic mode (GCPL) were performed. In the potential region of 2−1.5 V *vs.* Li/Li^+^ (corresponding to approximately 0−10 mAh·g^−1^ in the *E*−*C* curve), the original Fe_1+δ_Se structure exhibits no measurable alterations (Fig. 4a,b). The intensity and position of the *101* reflection remains apparently stable (taking into account experimental errors), but the *001* reflection broadens and its center of gravity shifts to slightly lower angles (Fig. 4c,d). Concordantly, the shape of the *E*−*C* curve is typical for a solid solution intercalating process involving a single phase. Further decrease of the potential (1.5−0.7 V *vs.* Li/Li^+^, which corresponds to 10−400 mAh·g^−1^ on the *E*^*−*^*C* curve) results in the rapid decomposition of Fe_1+δ_Se monitored by the drastic decrease in the intensities of both the *001* and *101* reflections (Fig. 4a). No new diffraction maxima are observed. The *E*−*C* curve reveals an almost flat plateau in this potential region, which unambiguously indicates the two-phase state of the system in accordance with reaction (1).

The results above can be summarized in a scenario for the electrochemical processes of a polycrystalline Fe_1+δ_Se electrode. In agreement with earlier results^11^, the major process favored by thermodynamics is the decomposition and complete reduction of Fe_1+δ_Se to metallic Fe. Electrochemical data together with *in-situ* PXRD measurements unveil that at higher potentials (*ca.* 1.7 V *vs.* Li/Li^+^) a subtle redox process takes place. This process conserves the initial structure topology and leads to the formation of a ternary phase Li_*x*_Fe_1+δ_Se with slightly larger lattice parameter *c* compared with the one of Fe_1+δ_Se. The potential increase which is observed after turning-off the electrochemical cell can be interpreted as spontaneous decomposition of the ternary phase due to its instability (cf. below). The small lattice parameters variation together with the small charge transfer indicates the insertion of a tiny amount of Li^+^ which leads to the formation of the phase Li_*x*_Fe_1+δ_Se with preserved structure topology. However, studies of bulk Li_*x*_Fe_1+δ_Se are hampered by its extreme instability and the overlap of intercalation and decomposition processes in the narrow potential region.

We assumed that the use of Fe_1+δ_Se crystal aggregates instead of a polycrystalline electrode can kinetically inhibit the decomposition process. Polycrystalline electrodes consist of a large amount of relatively small crystallites (1–10 μm in size), crystal aggregates contain several single crystals of much larger size (100–1000 μm). Surface effects (including the decomposition of Li_*x*_Fe_1+δ_Se and the formation of metallic Fe) in the latter case should be much less pronounced. Furthermore, the application of crystals allows avoid the use of carbon black and polyvinylidenfluorid in the measurement cell that makes the corresponding electrochemical side reactions negligible. The electrochemical behavior of Fe_1+δ_Se crystal agglomerates differs only slightly from that of the polycrystalline electrodes (Fig. SI5). The differences mainly concern a shift of the redox peaks to higher potential values. The first reduction peak, which corresponds to the suggested intercalation process, appears at *ca*. 1.8 V *vs*. Li/Li^+^. It is followed by the major peak (attributed to the conversion reaction) with an onset at *ca*. 1.7 V. This major peak is distorted in the CVA curve because the drastic volume change causes the destruction of the crystal. *I*–*t* curves obtained by long-time potentiostatic experiment at 1.7 V are close to those of the polycrystalline material. The current (in module) increases in the course of the 30 hours experiment. The amount of charge passed through the cell before the current increases corresponds to *ca.* 0.06 Li^+^ per f.u.

**Figure 5:**
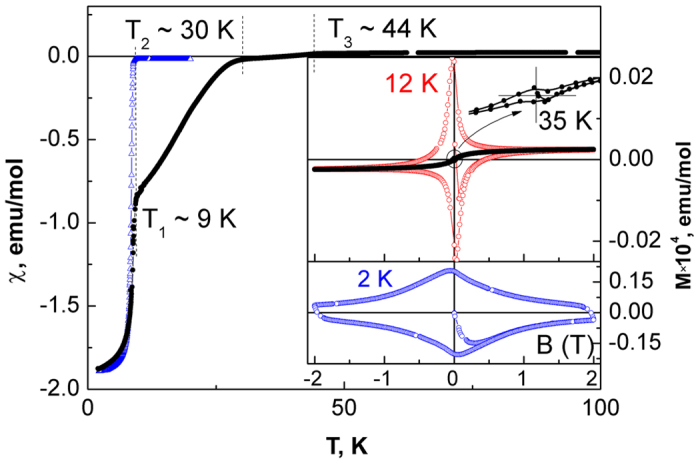
Temperature dependence of the magnetic susceptibility of initial Fe_1+δ_Se crystal aggregates (open blue triangles) and that after treatment at 1.7 V vs. Li/Li^+^ (solid black circles) measured in the ZFC regime at 0.01 T. The insets show the field dependence of magnetization at temperatures below the superconducting transition measured at 12 K and 35 K (top) and 2 K (bottom).

Magnetic measurements of the Fe_1+δ_Se crystal aggregates after treatment at 1.7 V indicate the presence of inhomogeneous superconducting phases. The temperature dependence of the magnetic susceptibility (Fig. 5) demonstrates a strong diamagnetic response (Meissner effect) at low temperatures corresponding to 100% volume fraction of the superconducting phases. At higher temperatures the χ(*T*) curve shows three kinks at *T*_1 _~ 9 K, *T*_2_ ~ 30 K and *T*_3_ ~ 44 K. It becomes positive in the range *T*_2_ < *T* < *T*_3_. The *T*_1_ anomaly can be attributed to the superconducting phase transition in binary (tetragonal) Fe_1+δ_Se which predominates in the studied sample. The magnetization curve measured at 2 K (lower inset to Fig. 5) is typical for Fe_1+δ_Se single crystals^17^. Two other anomalies can be attributed to two new superconducting phases. Some confirmation of the superconducting nature of the phases in the region *T*_1_ < *T* < *T*_3_ can be found in magnetization curves measured at 12 K and 35 K (upper inset to Fig. 5) indicating a small diamagnetic response. From the results of the chronopotentiometric experiment (Fig. SI6) we assume that in the products of the electrochemical treatment of the Fe_1+δ_Se crystal aggregates there are single-phase domains with slightly different content of Li.

The enhancement of *T*_c_ observed for the Fe_1+δ_Se crystal after electrochemical treatment confirms the suggested formation of Li_*x*_Fe_1+δ_Se. As additional technique to determine the amount of Li^+^, ICP-MS analysis was performed for polycrystalline electrodes (Table SI1). The highest Li content was detected in the electrodes subjected to the LVS followed by rather long CA at 1.55 V *vs*. Li/Li^+^. The relative Li-content in the electrode subjected to the intercalation reaction conditions (PITT with CA at 1.7 V) can be estimated as 0.06 Li per f.u. Fe_1+δ_Se. The result is in perfect agreement with the electrochemical data but this agreement merely reflects the tendencies for the different redox processes since capacity values are strongly sensitive to experimental conditions like the distribution of Fe_1+δ_Se particles in the electrode active layer and its thickness.

**Figure 6:**
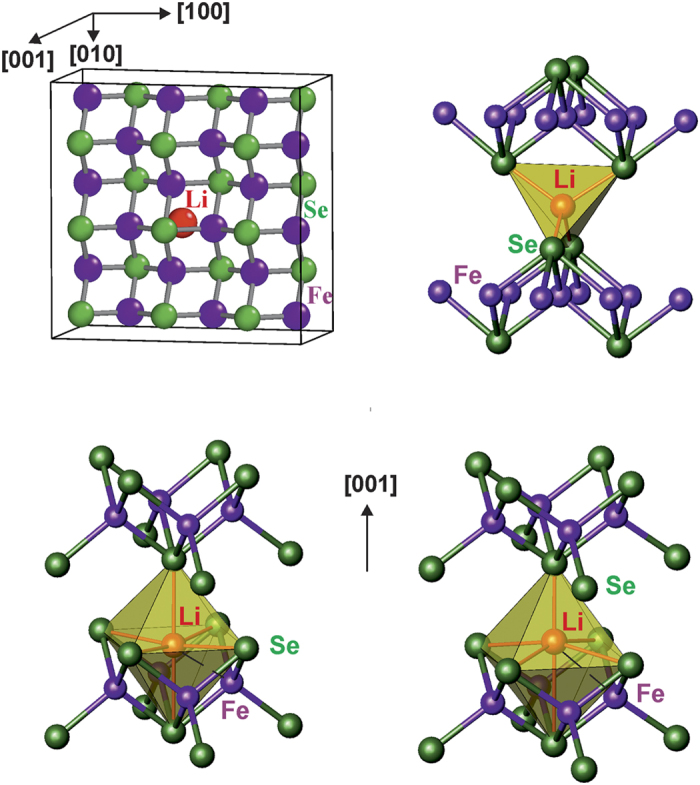
The optimized 3 × 3 supercell of the tetragonal structure of Li_1/18_FeSe (left top; the 4-fold symmetry around Li (red) is essentially preserved, relaxation of Fe (purple) and Se (green) is taking place basically for nearest neighbors, only) with Li filling tetrahedral voids (right top) and octahedral ones (ideal–left bottom, optimized–right bottom). Relaxing all atoms with respect to the total energy shifts Li off-center and reduces the undulation of the Fe-Se layers (right bottom).

*In-situ* PXRD data together with the magnetic measurements indicate bulk (i.e. not surface) formation of the superconducting phase which is structurally similar to tetragonal Fe_1+δ_Se. In case of tetragonal Fe_1+δ_Se, such a *T*_c_ increase is mainly achieved by electron doping^4^,^5^,^6^,^7^. The electrochemical insertion of Li into the Fe_1+δ_Se structure can be considered as one possible way of electron doping. Due to the close-packed features of the Fe_1+δ_Se structure (defect anti-fluorite blocks), there are tetrahedral and distorted octahedral voids (Fig. 6). For tetrahedral coordination, the calculated Li−Se distances (*ca.* 2.28 Å) are significantly shorter than those in the Li_2_Se structure (2.6 Å)^18^. For octahedral voids, the estimated Li−Se distances (*ca.* 2.57–2.95 Å) are comparable to the ones in Li_2_Se, while the Li−Fe distances (*ca.* 2.39 Å) are too short. However, shift of the Li in [001] direction results in a square-pyramidal coordination of Li with longer Li−Fe contacts.

A similar effect is observed in LiFeAs (PbClF type)^19^, where the anti-PbO-like topology is combined with a square-pyramidal Li^+^ coordination. Here, Li cations stabilize the whole structure because of Fe^3+^/Fe^2+^ reduction. It should be noted that the size of the anti-fluorite [Fe_2_As_2_] layer in LiFeAs is very close to that of the [Fe_2_Se_2_] layer in Fe_1+δ_Se. Obviously, complete Li insertion into Fe_1+δ_Se would correspond to the complete reduction Fe^2+^/Fe^1+^, thereby limiting the amount of inserted Li.

The total energy for different model structures and Li concentration *x* was calculated to investigate the stability of Li_*x*_FeSe with Li atoms occupying different sites. From chemical experience, it is expected that Li occupies tetrahedral or octahedral voids between the Se atoms (Fig. 6). Without relaxation of Se coordinates, we find a binding energy of slightly more than 1 eV per Li atom occupying the octahedral void, essentially irrespective of the Li concentration (Table SI2). For the tetrahedral position, the binding energy is reduced considerably to less than 0.5 eV per Li. Due to this large energy difference and the large Li mobility, lithium occupation of the tetrahedral void can be safely discarded near the equilibrium. Relaxing all atoms without symmetry constraint (space group *P*1) in a Li_1/18_FeSe super cell (Fig. 6) and with a Li concentration close to the experimental value, the binding energy increases (Table SI2) by about 0.2 eV per Li. The predominant structural feature is an off-center Li position (Fig. 6), and a smoothing of the FeSe layers. The fourfold symmetry of the Li position and its environment however, is essentially preserved.

We also calculated the stability of Li_*x*_FeSe (*x* = 1/18 = 0.056, near the experimental Li content) with respect to its decomposition into Li_2_Se, Fe and FeSe. Li_1/18_FeSe is not stable and thus should, under equilibrium conditions, decompose towards FeSe, Li_2_Se and Fe with an energy gain between 0.20 eV (for non-magnetic Fe clusters) and 0.42 eV (for magnetic Fe clusters) per Li (Table SI2). This is in agreement with the available experimental data. The calculated decomposition energy is only slightly dependent on the magnetic state of Fe in the clusters formed during the decomposition. For the experimentally observed superparamagnetic iron clusters the energy should be in-between the two limiting cases.

**Figure 7:**
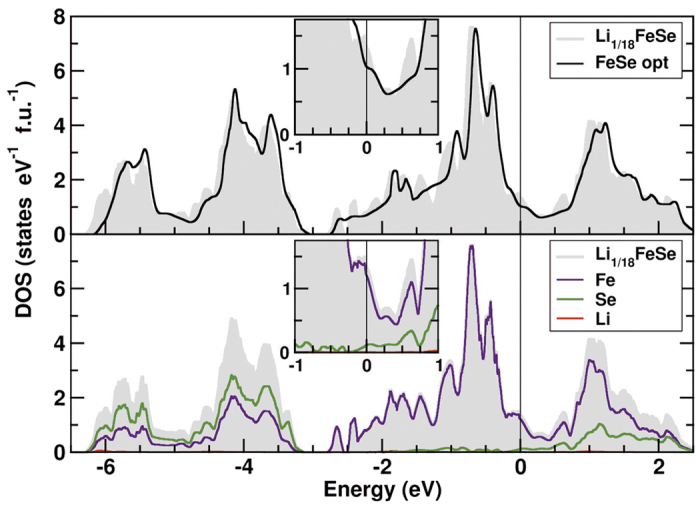
Total and partial electronic density of states (DOS) for Li_1/18_FeSe. The Fermi level *E*_F_ is at zero energy. Upper panel: Insertion of Li leads to a significant change of the electronic structure beyond a rigid band behavior (shift of *E*_F_, only) compared to the parent compound FeSe (with optimized Se position, see text), in particular to a sizeable increase of the DOS at *E*_*F*_ (insert). Lower panel: As in the idealized parent compound FeSe, the states near *E*_F_ originate almost exclusively from Fe with a small Se admixture; the Li-contribution is very small owing to the small Li concentration.

For a better understanding of the superconducting properties of Li_*x*_Fe_1+δ_Se, the change of the electronic density of states near the Fermi level *E*_F_ upon Li insertion is a crucial parameter. Comparison of the calculated DOS (Fig. 7 and Table SI2) for Li_1/18_FeSe and the parent compound FeSe yields three main results: (*i*) Surpr*i*singly, the insertion of Li leads to a sizeable re-structuring of the DOS which goes beyond the changes expected on basis of a rigid band picture and results in an increase of the DOS at *E*_F_. (*ii*) The total band width is basically unchanged. (*iii*) The main increase of the DOS at *E*_F_ originates from more distant Se and Fe neighbors and not from the closer Li environment. This demonstrates that the additional electron donated by the Li ion is rather delocalized. However, a possible formation of polarons (beyond the DFT calculations) might modify this picture.

The observed changes in the electronic structure evidence the formation of a ternary compound which is chemically different from Fe_1+δ_Se. The appearance of a DOS spike at the Fermi level is in line with the observed higher critical temperature of the superconductivity.

In summary, our results are in line with earlier findings that the Fe_1+δ_Se structure pattern has a strong tendency to preserve the electron count of the [Fe_2_Se_2_] layers. Thus, the homogeneity range of these structure units with respect to heterovalent substitution or the insertion of electropositive metals is rather limited. Even in case of structure alterations which result in substantial increases of the *T*_c_ values, the charge of the anti-fluorite layer changes only marginally^7^,^8^. Our finding suggests that the significant *T*_c_-enhancement of Fe_1+δ_Se can be achieved by a merely minute reduction of the Fe cations without increasing the [Fe_2_Se_2_]-interlayer separation. Therefore, it appears promising to investigate if the carrier concentration (or the iron valence) influences the *T*_c_-value of layered iron-based selenides to a much larger extent than the interlayer separation.

## Methods

### Preparation

Polycrystalline sample of Fe_1+δ_Se was prepared under argon atmosphere using iron pieces (99,995%) and selenium shots (99,999%). Glassy carbon crucibles with a lid were filled with Fe/Se mixture with molar ratio close to 1 : 1 (Fe : Se = *z* : 1 and 0.98 ≤ *z* ≤ 1.02), placed into vacuumed quartz ampoule and subjected to high-temperature annealing (for details see ref. 13). Large crystals of Fe_1+δ_Se were prepared using the KCl/AlCl_3_ flux at 427 °C in evacuated quartz ampoules in constant temperature gradient^20^.

### Basic characterization

Microstructure investigation of polycrystalline samples and crystals was performed with JEOL JSM 5510 (LaB_6_ cathode, 30 kV) scanning microscope. Phase identification and lattice parameters determination (for polycrystalline sample and crystals) were performed using room-temperature X-ray powder diffraction data obtained by image plate Guinier camera Huber G670, (Co*K*_α1_ radiation, *λ* = 1.78892 Å). Crystalline Ge (*a* = 5.6576 Å, ref. 21) was used as internal standard. *Ex-situ* X-ray diffraction data for polycrystalline electrodes were collected in air using Bruker D8-Advance diffractometer (Cu*K*_α1_ radiation, *λ* = 1.540598 Å, LynxEye PSD) in reflection mode. The electrodes previously were covered by air-protective one side sticky tape.

### Electrochemical processing

Electrochemical treatments were performed in two-electrode setup with metallic Li as counter and reference electrodes. The commercial electrolyte (1 mol L^−1^ LiPF_6_ in a mixture of dimethyl carbonate and ethylene carbonate (1: 1 by volume, Merck) was used. The glass fiber was applied as separator. Working electrode (in case of polycrystalline samples) was prepared by screen printing the slurry containing the active material, carbon black and PVDF (mass ratio 85 : 5 : 10) in N-methylpyrrolidone onto Al-substrate. Load of active material was approx. 1 mg·cm^−2^. In a case of Fe_1+δ_Se crystals, one crystal was placed by its flat face between current collector and separator directly. Electrochemical cells were assembled and disassembled in Ar atmosphere. Potentiostat/galvanostat Biologic VMP-3 was used for data collecting. Several electrochemical techniques were applied: linear and cyclic voltammetry (LVS and CVA) between 1.0 V and 2.5 V *vs.* Li/Li^+^ with scan rate of 0.02 mV·s^−1 ^and 0.05 mV·s^−1^; potentiostatic intermittent titration technique (PITT) with 0.01 V step in combination with chronoamperometry (CA) at selected potentials; and galvanostatic charge with potential limitation (GCPL) at current density of approx. 20 mA·g^−1^.

### *In-situ* powder X-ray diffraction

The experiment was carried out on polycrystalline electrodes. The two-electrode electrochemical cell was analogous to the mentioned above but with Be window at the anode side and porous polypropylene separator (“UFIM”, Russia) instead of glass fiber. The diffraction data were collected using Bruker D8-Advance diffractometer (Cu*K*_α1_ radiation, *λ* = 1.540598 Å, LynxEye PSD) in reflection mode. During the experiment, a constant current of −15 μA (approx. 20 mA·g^−1^) was applied at the working electrode; diffraction patterns were collected every 30 min until the potential decreased to 0.7 V *vs*. Li/Li^+^ and the electrochemical cell was switched off.

### Magnetic measurements

Magnetisation was measured by vibration magnetometer (PPMS-9T, Quantum Design) applying fields of 10–1000 Oe. The specimens (polycrystalline samples, polycrystalline electrodes, crystals aggregates) were put inside plastic holders and then placed into the magnetometer in Ar atmosphere.

### Chemical analysis

The lithium/iron ratio in electrochemically treated electrodes was analyzed by ICP-MS by means of ICP-MS spectrometer ELAN DRC II.

### Band structure calculations

Relativistic density functional (DFT) electronic structure calculations were performed using the full-potential local-orbital FPLO code^22^,^23^ (version fplo14.00-47). For the exchange-correlation potential, within the local density approximation (LDA) and the general gradient approximation (GGA), the parametrization of Perdew-Wang^24^ and Perdew-Burke-Ernzerhof^25^ were chosen, respectively. To obtain precise total energy and band structure information, the calculations were carried out on well converged meshes of up to 3888 *k* points depending on the cell volume. For all calculations, the respective experimental lattice parameters have been used.

## Additional Information

**How to cite this article**: Alekseeva, A. M. *et al*. New superconductor Li_*x*_Fe_1+δ_Se (*x* ≤ 0.07, T_c_ up to 44 K) by an electrochemical route. *Sci. Rep.* **6**, 25624; doi: 10.1038/srep25624 (2016).

## Supplementary Material

Supplementary Information
