# Supplementary material for: New superconductor LixFe1+δSe (x ≤ 0.07, Tc up to 44 K) by an electrochemical route
Source: Sci Rep. 2016 May 11;6:25624. doi: 10.1038/srep25624 (PMC4863254; doi:10.1038/srep25624)
Supplement: Supplementary Information [file srep25624-s1.doc]

***Supporting Information***

**New superconductor Li_x_Fe_1+δ_Se (x ≤ 0.07, T_c_ up to 44 K) by an electrochemical route**

**Anastasia M. Alekseeva^1,*^, Oleg A. Drozhzhin^1,5^, Kirill A. Dosaev^1,5^, Evgeny V. Antipov^1,*^, Konstantin V. Zakharov^2^, Olga S. Volkova^2^, Dmitriy A. Chareev^3^, Alexander N. Vasiliev^2^, Cevriye Koz^4^, Ulrich Schwarz^4^, Helge Rosner^4^, and Yuri Grin^4,*^**

^1^Department of Chemistry, Lomonosov Moscow State University, 119991 Moscow, Russia

^2^Department of Physics, Lomonosov Moscow State University, 119991 Moscow, Russia

^3^Institute of Experimental Mineralogy, Russian Academy of Sciences, 142432 Chernogolovka, Russia

^4^Max-Planck-Institut für Chemische Physik fester Stoffe, 01187 Dresden, Germany

^5^MPG-MSU Partner Group, Department of Chemistry, Lomonosov Moscow State University, 119991 Moscow, Russia

**^*^To whom the correspondence may be addressed:** [**alekseevaam@gmail.com**](mailto:alekseevaam@gmail.com)**; evgeny.antipov@gmail.com;** [**grin@cpfs.mpg.de**](mailto:grin@cpfs.mpg.de)**.**

**Table SI1**. The results of ICP-MS analysis (element concentrations, molar ratio) of the polycrystalline Fe_1+δ_Se electrodes

| *Electrochemical treatment* | *c*_Li_,  μg⋅l^−1^ | *c*_Fe_, (^54^Fe) μg⋅l^−1^ | *c*_Fe_, (^57^Fe), μg⋅l^−1^ | *n*_Li_/*n*_Fe_ | Δ*n*_Li_/*n*_Fe_^1)^ |
| --- | --- | --- | --- | --- | --- |
| Impregnated with electrolyte^2)^ | 40 ± 3 | 2200 ± 300 | 2200 ± 300 | 0.140±0.02 | − |
| PITT (2.8–1.7 V *vs*. Li/Li^+^) followed by CA at 1.7 V for 3 days | 60 ± 5 | 2100 ± 300 | 2100 ± 300 | 0.2204±0.03 | 0.08±0.05 |
| LVS (0.02 mV⋅s^−1^, 2.8–1.55 V *vs*. Li/Li^+^) followed by CA at 1.55 V for 1 day | 120 ± 10 | 1700± 200 | 1700 ± 200 | 0.5445±0.04 | 0.4045±0.06 |
| CVA (0.05 mV⋅s^–1^, 1.3–2 V *vs*. Li/Li^+^), 3 cycles | 100 ± 8 | 1800 ± 300 | 1800 ± 300 | 0.4286±0.08 | 0.2886±0.10 |

^1)^Relative lithium content after subtraction of lithium impregnated with liquid electrolyte (electrode was put in the liquid electrolyte for half an hour).

^2)^Initial polycrystalline electrode impregnated with electrolyte.

**Table SI2**. Stability of Li_x_FeSe. Results of the quantum chemical calculations and low-temperature experiments.

**Energies** (in Hartree per formula unit, 1 Hartree ≈ 27.21 eV)**:**

Compound LDA_exp LDA_opt GGA_exp GGA_opt

(exp = no internal relaxation, opt = internal relaxation with respect to total energy, n.a. for no internal degrees of freedom)

Li -7.40941 n.a. -7.52139 n.a.

Fe (bcc, fm) -1270.580879 n.a. -1272.805328 n.a.

Fe (bcc, nm) -1270.564390 n.a. -1272.784696 n.a.

(nm = non-magmetic, fm = ferromagnetic)

Li_2_Se -2442.048295 n.a. -2445.418591 n.a.

FeSe -3697.677350 -3697.684749 -3703.049246 -3703.052754

LiFeSe oct -3705.135811 -3710.600307

LiFeSe tet -3705.112246 -3710.575194

(oct = Li in octahedral void; tet = Li in tetrahedral void)

Li_1/2_FeSe -3701.409183 -3701.409386 -3706.825433 -3706.826328

Li_1/18_FeSe -3698.099001

**Stability:**

LiFeSe (oct) ⇦ FeSe + Li E_B_=-1.33 eV /Li (LDA) -0.81 eV / Li (GGA)

LiFeSe (tet) ⇦ FeSe + Li E_B_=-0.69 eV /Li (LDA)

Li_1/2_FeSe ⇦ FeSe + ½ Li not optimized E_B_=-1.47 eV /Li (LDA) -0.84 eV / Li (GGA)

optimized E_B_=-1.08 eV /Li (LDA) -0.70 eV / Li (GGA)

Li_1/18_FeSe ⇦ FeSe + 1/18 Li optimized E_B_=-1.28 eV /Li (LDA)

(⇒Li_1/18_FeSe forms with a formation energy gain of 1.28 eV per Li from FeSe and Li)

Li_1/18_FeSe ⇦ 35/36 FeSe + 1/36 Li_2_Se + 1/36 Fe

optimized (Fe fm) E_B_= 0.42 eV / Li (LDA)

optimized (Fe nm) E_B_= 0.20 eV / Li (LDA)

(⇒Li_1/18_FeSe decomposes this way with a decomposition energy of 0.20 (0.42) eV per Li)

We performed some experiments at reduced temperatures. The results of the magnetic measurements for the polycrystalline sample electrochemically treated at -20 ^o^C reveal the decreasing of the amount of either metallic iron and Li_x_Fe_1+δ_Se phases. Therefore, reduced temperatures may stabilize the Li_x_Fe_1+δ_Se phase, but drastically decrease the rate of Li^+^ solid diffusion.


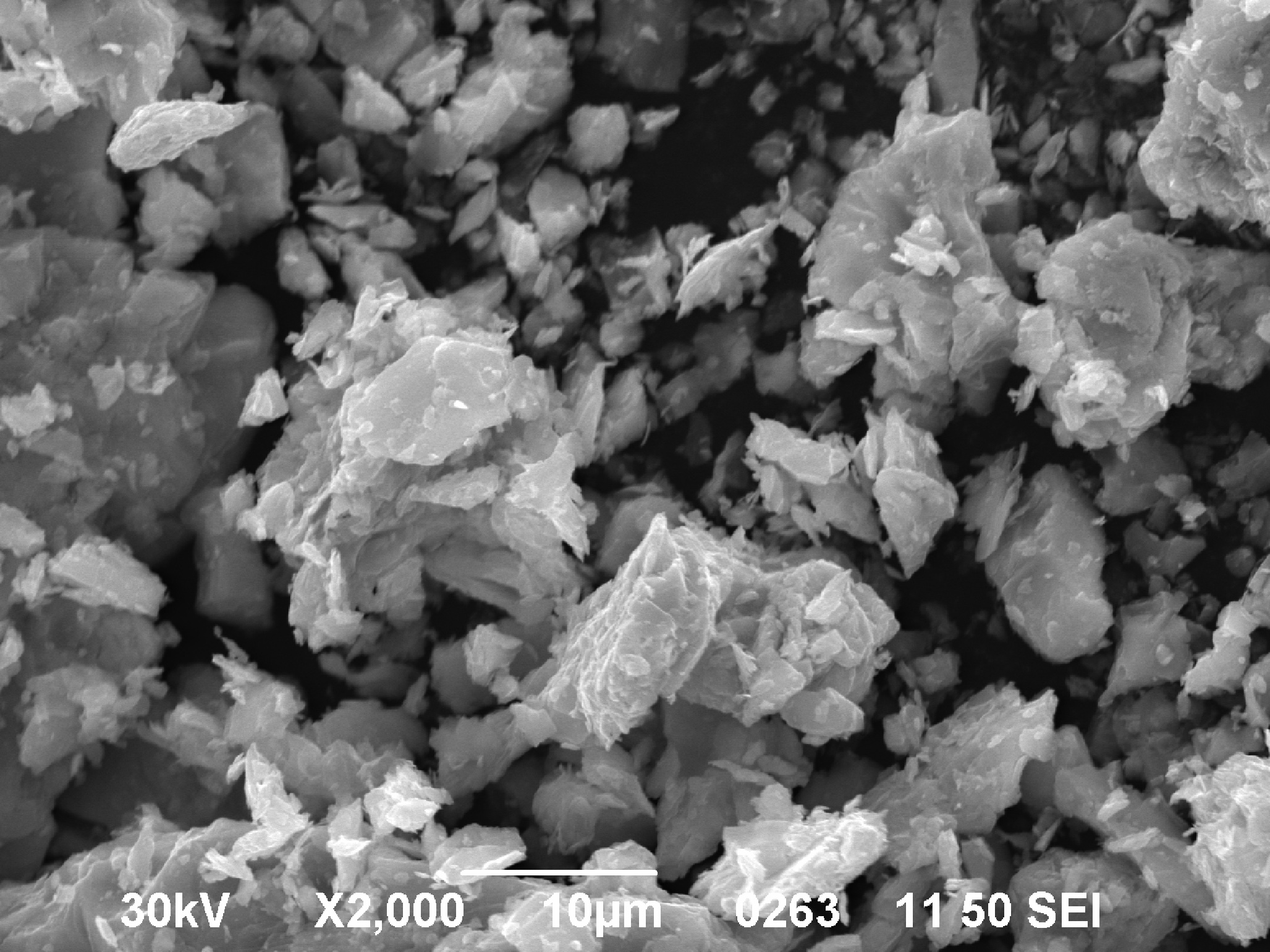


**Fig. SI1.** SEM image of the Fe_1+δ_Se polycrystalline single-phase sample.


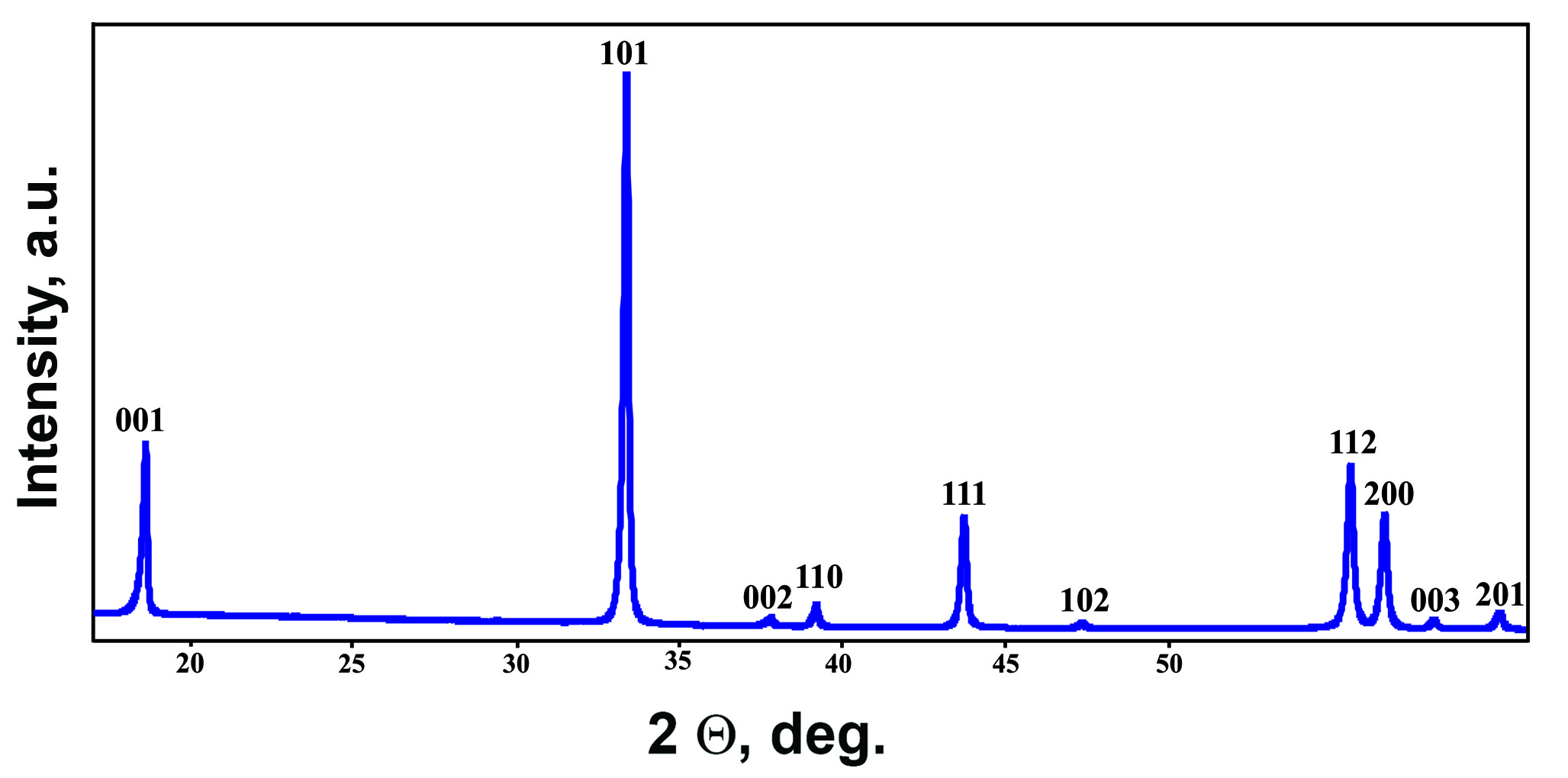


**Fig. SI2.** A part of X-ray powder diffraction pattern for Fe_1+δ_Se polycrystalline single-phase sample. Reflections of the tetragonal Fe_1+δ_Se are indexed.


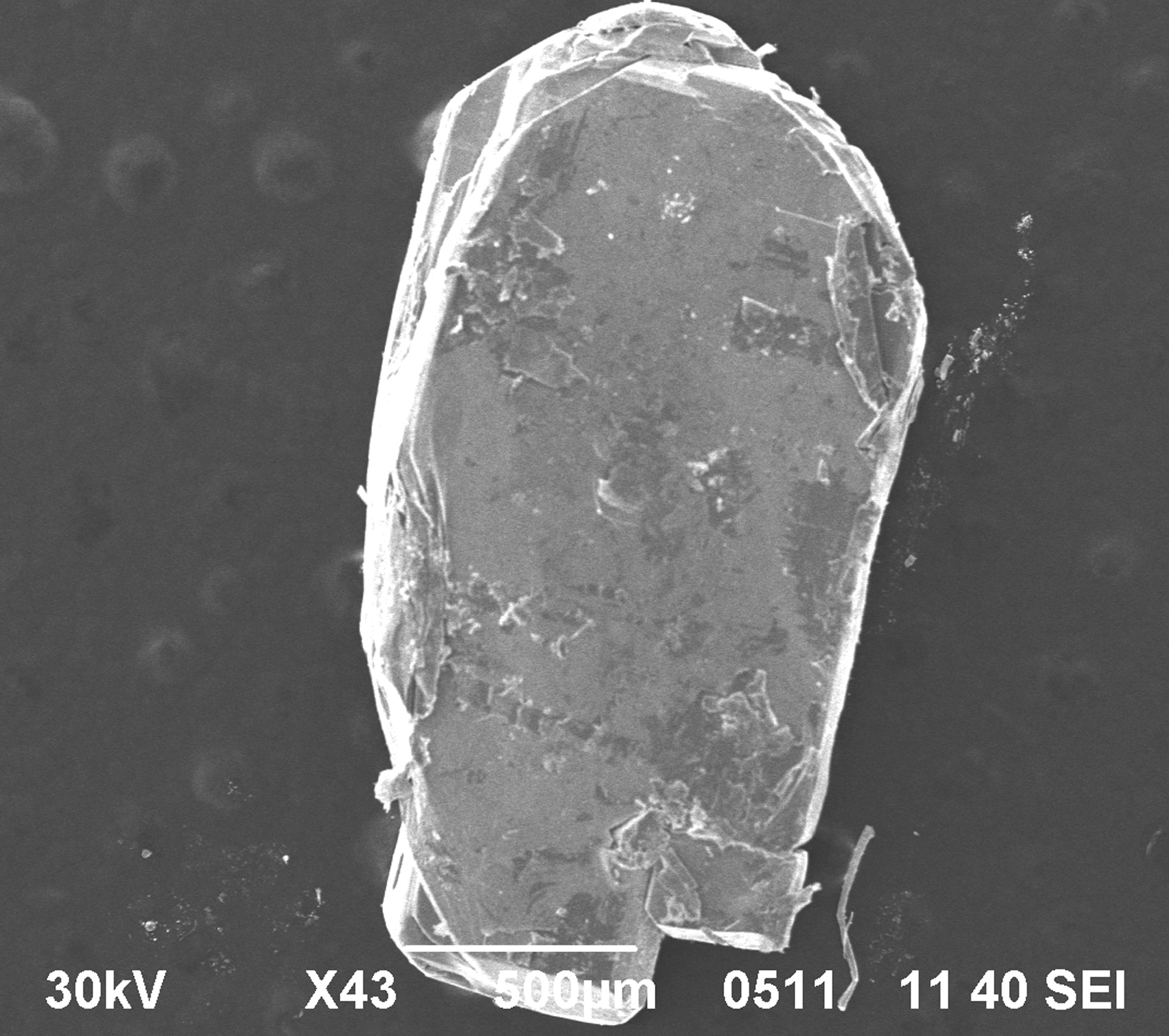


**Fig. SI3.** SEM image of Fe_1+δ_Se large crystal aggregate.


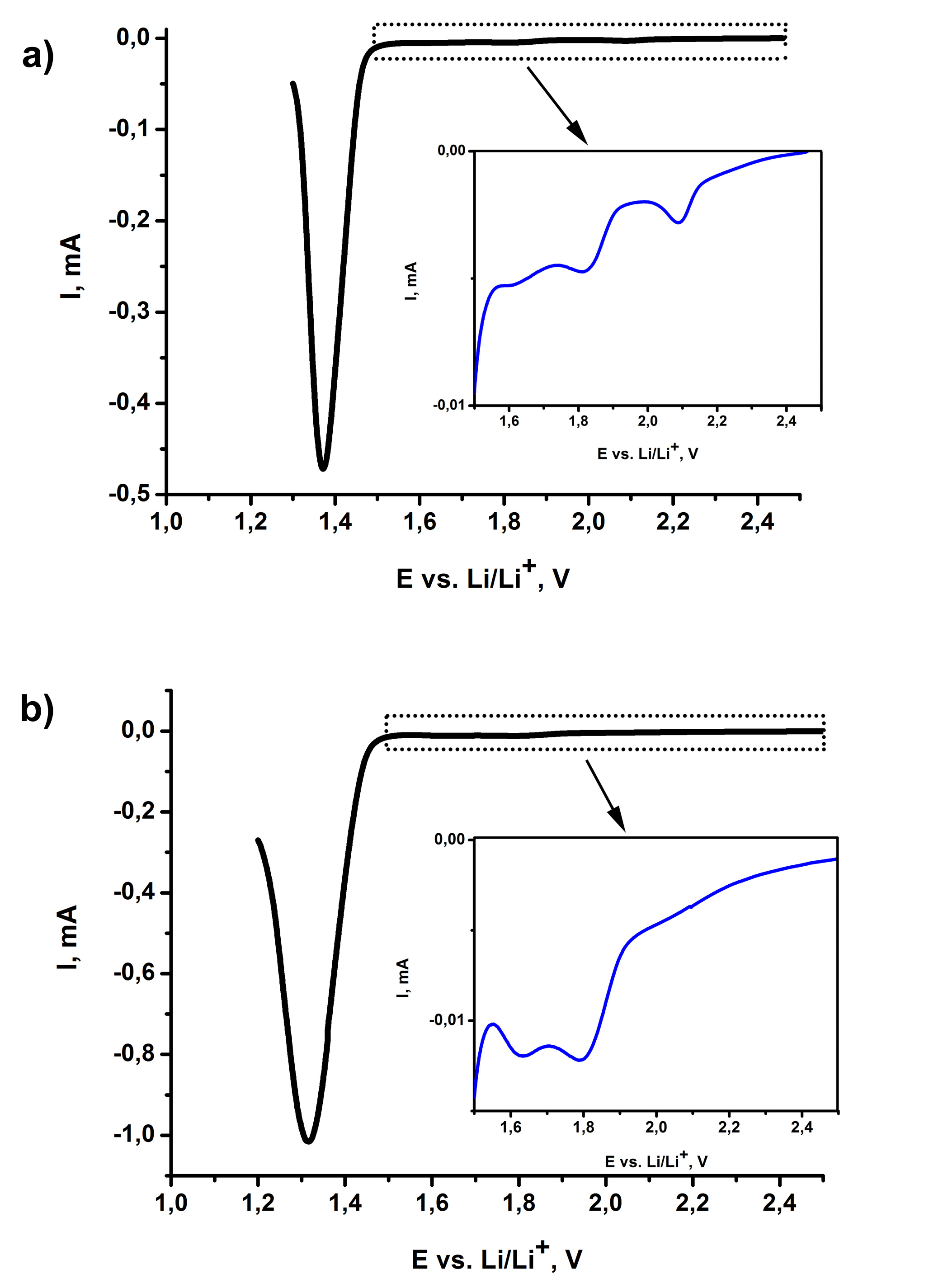


**Fig. SI4.** LVS (scan rate of 0.02 mV⋅s^−1^, 2.5−1.2 V *vs*. Li/Li^+^) curves for the polycrystalline Fe_1+δ_Se electrodes: **(*a*)** carbon-containing electrode; **(*b*)** carbon-free electrode.


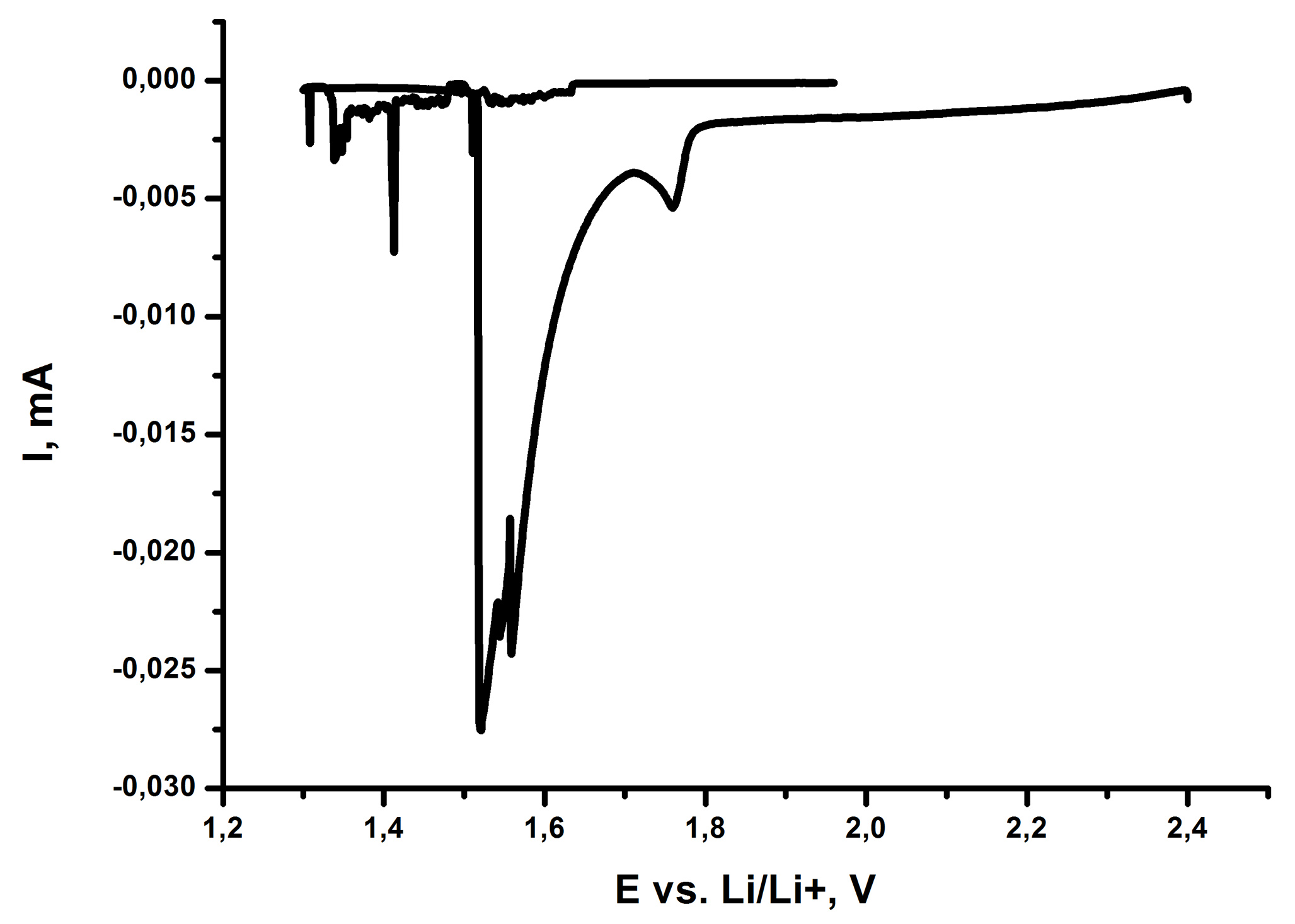


**Fig. SI5.** CVA curve for the crystal aggregate of Fe_1+δ_Se in potential range 1.2−2.5 V *vs*. Li/Li^+^ (scan rate of 0.02 mV⋅s^−1^).


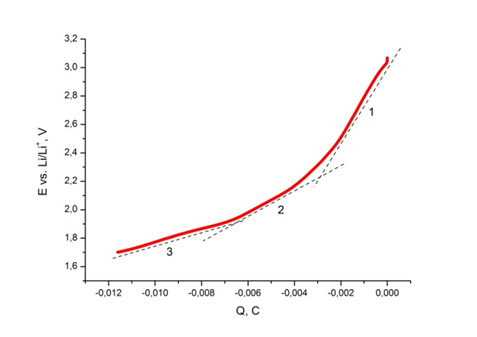


**Fig. SI6.** E-Q dependence for the chronopotentiometric experiment on the “oriented crystal” of Fe_1+δ_Se. Two-electrode cell, Li as a counter electrode, 1M LiPF_6_ in EC:DMC = 1:1 as electrolyte, I = -1 mkA. The behavior of Fe_1+δ_Se polycrystalline electrodes during in situ XRPD or PITT measurements indicates single-phase (solid solution) type of the intercalation process producing a concentration gradient of Li^+^ inside the single particle during electrochemical experiment. However, after turning off the electrochemical cell the relaxation of this gradient should be rather fast. On the other hand, the shape of E-Q or I-E curves at the 3.0-1.7 V vs. Li/Li^+^ region shows that the process is not an “ideal” formation of single solid solution, but a combination of several solid solutions with slightly different shapes of intercalation isotherms (and, apparently, crystal structure parameters). The presented E-Q curve (charge of the “oriented crystal” with 1 mkA constant current) contains at least three regions with different slopes. Therefore the most probable reason for the observed values of T_c_ (30 and 44 K) is the presence of the single-phase domains with slightly different x in Li_x_Fe_1+δ_Se inside one “oriented crystal” (in addition, obviously, x is larger near the surface and lower in the center of the particle).
